# Successful Ageing in Singapore

**DOI:** 10.3390/geriatrics3040081

**Published:** 2018-11-19

**Authors:** Andrew Rogerson, Simon Stacey

**Affiliations:** Department of General Medicine, Medical Centre Level 9, Sengkang General Hospital, 110 Sengkang East Way, Singapore 544886, Singapore; Simon.stacey@singhealth.com.sg

**Keywords:** successful ageing, positive ageing, gerontology, Singapore, frailty, frailty screening

## Abstract

Singapore has experienced rapid development in the past 50 years. This has presented unique challenges with regard to land space and a rapidly ageing population. The role of extrinsic factors in successful ageing is well documented, and places a degree of responsibility on the state and healthcare systems. Singapore has taken many proactive measures to meet this responsibility by implementing policy changes across multiple domains including housing, transport, education and research. One hospital in the north east of Singapore has undertaken a frailty screening program that aims to identify, prevent and reverse frailty at an early stage. This paper provides a review of these national and regional measures.

Singapore is a global business hub and a cultural melting pot. Situated at southernmost tip of continental Asia, it played a key role in historical East-West trade routes. As such it experienced high rates of immigration from other Asian countries, especially China. After gaining independence in 1965 it enjoyed exceptionally high economic growth under the leadership of its visionary founding father Lee Kuan Yew. It progressed from developing to developed country status in a single generation, becoming one of the four “Asian Tiger” economies, and is now one of the wealthiest and most technologically advanced countries in the world. Its population currently sits at just over 5.6 million, three-quarters of which are of Chinese descent. The remainder are predominantly of Malay (13%) or Indian (9%) descent [1]. This ethnic diversity gives rise to a wide range of languages, religions and cultures within an island city-state only 50 km long and 27 km wide. The country has four official languages—English, Mandarin, Malay and Tamil—but there exists a plethora of Chinese dialects, other Indian languages, and creolised versions of Portuguese and English.

## 1. Attitudes to the Elderly in Singapore

Globally, links between economic growth and negative attitudes to the elderly have been proposed but not conclusively proven [2]. Given its rapid growth, one might expect such an effect in Singapore. This may however be somewhat mitigated by the traditional Asian values of filial piety and a deep respect for elders. A 1988 survey of university students found no significant difference in favourability indices between young and old [3]. A 2014 questionnaire study of 3000 older Singaporeans [4] found that although 70% felt that elders were respected, discrimination does exist—especially towards working older people. Elders of lower educational level were more likely to hold negative views of the young, and feel uncomfortable conversing with younger people. This may reflect the large generational differences in educational levels typical of rapidly advancing societies. In keeping with this, the survey also found that older Singaporeans are highly motivated to continue lifelong learning, to support their management of everyday life, keep up with changes, and understand themselves and others better.

## 2. Singapore’s Challenges and the Role of Public Policy

While the challenges of an ageing population are global, Singapore contends with disproportionately high rates of population ageing [5] as well as land space limitations. Currently around 25%, the proportion of over-65s is projected to double by 2030 [6]. This brings a unique set of challenges, which is being tackled head-on through governmental policy and urban planning.

The concept of successful ageing arose in the 1950s, but to date there is no universally agreed definition. It is an evolving and controversial concept. The work of Rowe and Kahn was strongly influential in its development. In 1987 they introduced the idea of two different modes of disease-free ageing—“usual” and “successful” [7]. The latter was described in terms of three main components: avoiding disease and disability, high cognitive and physical function, and engagement with life. This idea was expanded through the MacArthur Research Network on Successful Aging Community Study—a cohort study of high-functioning older Americans conducted between 1988 and 1995 [8,9,10]. This study and others found that lifestyle factors such as exercise, diet, education, social support, and engaging in productive activities can significantly reduce disease and mortality risk, and limit (or even reverse) cognitive, physical and functional decline. The role of religion, spirituality and/or belief in promoting positive ageing is now also recognised [11].

Until the latter half of the 20th century it was thought that little could be done to modify the course of ageing, and that genetic factors were chiefly responsible for health in later life. The MacArthur study helped highlight behavioural influences on the ageing process and advanced the notion that older people are valuable and benefit from societal engagement. It catalysed a major shift in the study of gerontology and helped shape social policy by justifying the funding of studies and social programs [12,13].

The Rowe and Kahn model has received some criticism for several reasons: perceived over-reliance on biomedical aspects and its exclusion of individuals with chronic disease [14]; the negative implication of “unsuccessful” ageing within the terminology itself; and the fact that it excludes relatively fixed extrinsic factors in the ageing process. Even its political undertones have been questioned [15]. Moreover, the prevalence of self-defined successful ageing is much higher than that as defined by Rowe and Kahn’s model [16]. Other successful ageing models include “selective optimization with compensation” [17], (which advocates the optimization of function and compensation for loss of function within a targeted and individualized selection of specific life domains) and the “preventive and corrective proactivity” model [18] (which incorporates both the preventive/biomedical aspects of the Rowe and Kahn model and the adaptive approach of selective optimization with compensation). Despite controversies the concept persists and it remains one of the most intensely researched aspects of gerontology.

It is also now recognised that although the ageing process is to some extent self-determined, it is also influenced by many fixed structural factors throughout the life course [19,20]. These include: the influence of social contacts on health behaviours; historical context with its attending disease burden; the effect of geography on economic factors, cultural perceptions of ageing and health behaviour; and social factors such as race, gender, educational level, and financial adequacy.

These extrinsic factors highlight the role of public policy in successful ageing. In addition, the framing of frailty as a public health condition in the recent World Report on Ageing and Health calls for greater efforts aimed at promoting or maintaining the intrinsic capacity of people as they age. Singapore takes its ageing population seriously and is taking a comprehensive and active approach to addressing these needs.

## 3. National Measures

In 1982 the Ministry of Health (MOH) set up the Committee on the Problems of the Aged [21,22]. Its recommendations included the role of families in elder care; multigenerational living in public housing development; and the incentivisation of continued societal and economic contributions of the elderly. A “National Survey of Senior Citizens in Singapore” was conducted in 1995. The resulting 1999 report recognised the role of individual, family, community and state in elder care, and introduced the concept of “ageing-in-place”, which has guided subsequent policies until the present day.

Since then several committees have evolved under MOH direction—the latest of which is the Ministerial Committee on Ageing (2007). They began a “City For All Ages” project in 2011, publishing an “Action plan for successful ageing” in 2015 [23]. This plan emerged following a 12-month enquiry into the views of Singaporeans on positive ageing. Public opinion was explored through focus groups, online consultations and public “listening points”. It found that older Singaporeans’ wishes include:

- to remain employable in later life to maintain activity levels and financial independence
- to be involved in voluntary work
- educational opportunities for personal development, employability and maintenance of health
- interaction with society and respect from younger people
- access to good local elderly care centres and community medical care
- where possible, independence from their families through senior-friendly housing, transport, and public spaces
- Singapore-based ageing research

Initiatives and targets, to be achieved within 10–15 years, were drawn up across these domains. Some examples are detailed below.

### 3.1. Employment

Singapore already has one of the highest old-age employment rates within the OECD (Organisation for Economic Cooperation and Development). This will be improved further by: increasing the re-employment age from 65 to 67; offering grants to incentivise the creation of elder-friendly work processes and workplace design; and subsidising work skills courses for over-40s (“SkillsFuture”).

### 3.2. Lifelong Learning

In addition to SkillsFuture, a network of institutions including voluntary welfare and community organisations will provide 30,000 learning spaces for older adults under the title “Silver Academy”.

### 3.3. Volunteerism

A “Silver Volunteer Fund” will be set up with aim of raising US $40,000 (50% government-funded, 50% public donations) to support community organisations in the recruitment of volunteers.

### 3.4. Communities of Care

A “Wellness Programme” will coordinate the creation of “Wellness Hubs” in various neighbourhoods city-wide. These will be located in community centres and provide social interaction, health education and exercise classes for seniors. In addition the Ministry of Health will facilitate the recruitment of volunteers to befriending services for the socially isolated, in at least 50 Singapore neighbourhoods.

### 3.5. Intergenerational Harmony and Respect for Seniors

The Housing Development Board (HDB) will continue to design housing that encourages seniors to live with/near to their married children, promoting intergenerational harmony. Elder and childcare services will be co-located within HDB developments. In addition, a “Passion Silver” Card will be introduced, which will encourage services to offer privileges to seniors.

### 3.6. Geriatric Medicine, Care and Research

The Ministry of Health has committed to improve medical care for seniors by bolstering inpatient and community geriatric services. There will also be marked increases in the number of Community Hospital and nursing home beds. In addition the National Research Foundation will reserve a $200 million budget dedicated to the facilitation of ageing research.

### 3.7. Transport and Urban Planning

The Land Transport Authority will take measures to promote ease of mobility around the city. This will include: the introduction of 35 “Silver Zones”, with traffic calming measures to promote safety; “Green Man Plus” pedestrian crossings with prolonged crossing times [24]; installation of lifts at specific pedestrian overhead bridges; wheelchair-friendly buses; and modifications to bus routes with regard to seniors’ travel priorities.

## 4. A Local Initiative in North Eastern Singapore

The World Health Organisation framework for healthy ageing [25] advocates the provision of access to older-person-centred and integrated care. In line with this, Sengkang General Hospital—the regional hospital for Singapore’s north east—has developed a complete care path that begins with the community functional screening of older adults, with subsequent triaging to stage-appropriate care. A central aim of this project, which is funded by a National Medical Research Council grant, is to maintain and improve functional fitness, and to prevent or delay frailty progression in older adults. It is premised on the potential modifiability of both frailty and functional fitness. Frailty is an age-associated state of diminished reserves that confers susceptibility to adverse health outcomes. Its potential reversibility may make it an ideal target for interventions to impact healthy ageing. Functional fitness, representing the opposite end of the frailty continuum, is associated with successful ageing [26], and is conceptualized as the physical capability needed to undertake normal daily activities, independently and without early fatigue [27].

The Sengkang region of Singapore is a relatively new estate with a young mean age. As such its population affords a prime target for such a novel intervention aimed at promoting healthy ageing from a life course perspective. In line with this, the programme is offered to all residents over the age of 55, with the aim of identifying risk factors in middle age, when age-related functional decline is in the very early stages.

### 4.1. Frailty Screening

Termed “Individual Physical Proficiency Test for Seniors (IPPT-S)”, this novel community-based programme aims to identify frailty in older adults through their participation in a multi-domain geriatric screen and physical fitness testing. To ensure accessibility and encourage participation, the IPPT-S has been developed as a mobile platform anchored at the void decks of public housing blocks, senior activity centres (SACs), senior care centres (SCCs) or community clubs (CCs). Although response rate, acceptability and cost-effectiveness are beyond the study’s scope, meaningful numbers of residents volunteered their participation. The platform is at each site for 2–3 consecutive weeks, and is managed by members of a multidisciplinary team of health professionals (geriatrician, rehabilitation physician, physiotherapist, dietitian) and trained volunteers. Yearly follow-up visits will be conducted at the same site. Return rates will be assessed at this point.

The multi-domain geriatric screen was modelled after the Rapid Geriatric Assessment [28], and includes frailty screening, as well as cognitive, psychological, and nutritional assessments, using a structured questionnaire administered by a trained interviewer. Frailty is scored using the well-validated 5-item FRAIL scale. Cognition is assessed using the locally validated modified Chinese version of Mini Mental State Examination (CMMSE), and mood using the Geriatric Depression Scale (GDS). Nutritional status is evaluated using the Mini Nutritional Assessment-Short Form (MNA-SF) questionnaire [29,30]. Participants are also assessed for social vulnerability through questions on socio-economic status and social support factors. Functional performance in activities of daily living (ADL) and instrumental ADL (IADL) is evaluated using the Barthel Index and Lawton and Brody’s scale respectively [31,32]. Data on falls and healthcare utilization in the past year is captured via standardized questions.

The physical fitness test battery was modified from the Senior Fitness Test [33], and includes measures of flexibility, strength and power, mobility, balance, agility and aerobic endurance. Grip strength (measured by JAMAR dynamometer) and gait speed (based on a 10 m walk at usual pace) are core components. Additional tests include the back scratch and chair sit-and-reach tests (testing upper and lower body flexibility respectively) [34,35]; the chair stand test (assessing lower body strength and power) [36]; the box and block test (to evaluate upper limb power and coordination) [37]; the Timed Up-and-Go Test (measuring dynamic balance) [38]; and the 6-min walk test (6MWT), which scores cardiorespiratory endurance.

The hour-long visit culminates with the participant receiving a personal booklet detailing performance and counselling on lifestyle interventions. Since frailty is often both overlooked by physicians and misconstrued by elderly as an inevitable consequence of ageing, the programme provides an important opportunity to raise public awareness of its symptoms and signs, which may in turn prompt early medical review and intervention.

The IPPT-S pilot phase (performed at 3 SACs) is now complete and the platform is being extended across SACs, SCCs and CCs in north eastern Singapore. Pilot data from 135 older adults revealed that 73.3% were robust, 25.2% were pre-frail, and 1.5% were frail. Adjusting for age and gender, depression (OR = 2.90, 95% C.I 1.05–7.90, *p* = 0.040) and malnutrition (OR = 6.07, 95% C.I 2.52–14.64, *p* < 0.001) were independently associated with pre-frailty/frailty. Pre-frail/frail older adults had significantly poorer performance in tests of upper and lower limb power, tandem and dynamic balance, as well as endurance.

### 4.2. Post-Screening Care

Screening efforts must be complemented by actionable care plans addressing individuals’ frailty-associated risk. The senior participants are thus triaged to stage-appropriate care based on their frailty state. Robust seniors are invited to attend two group education sessions led by a physiotherapist and dietitian, which include demonstration of exercises for frailty prevention, as well as lectures and games addressing nutrition, with the aim of promoting self-management.

Pre-frail elderly present higher risk than their robust peers for falls, hospitalization and disability [32], and are markedly more prevalent in Singapore than the frail elderly. Pre-frailty may represent the optimal moment at which to address the cascade, since these individuals in this cohort are more likely to revert to robustness [39,40], and may yield greater functional improvements [33,41] than the frail group. Thus, pre-frail seniors are offered enrollment into a 4-month group-based multi-component physical exercise programme in combination with a nutritional intervention. Group-based sessions have been shown to boost older persons’ motivation for participation and potentially address the social contribution to frailty and the ageing process [42], while allowing for efficient use of limited health resources. The exercise programme, conducted once-weekly at the same sites as the IPPT-S platform, is designed to challenge all major muscle groups with specific emphasis on strength training, in addition to gait, balance and endurance, to target the major frailty elements of weakness and gait deficit. The nutritional intervention comprises 6 dietitian-led education sessions promoting healthy eating habits with respect to guideline recommendations for protein, energy, Vitamin D [43]. Participants will be tracked for changes in their physical fitness performance as well as reversal from the pre-frail to robust state.

Finally, we are in the process of establishing our Geriatric Services Hub (GSH), which will facilitate the integration of community screening efforts with follow-up medical care, as seniors who are assessed to be physically frail or who screen positive for geriatric syndromes (such as cognitive impairment, depressed mood, falls) can be assured timely comprehensive geriatric assessment (CGA) [44]. GSH provides the suite of medical services necessary for the frail older adult in primary care, including CGA by geriatric-trained community nurses and family physicians, rehabilitation by community physiotherapists and/or occupational therapists based on the senior’s needs, individualized dietitian review, and access to medical social services as indicated. Monthly multi-disciplinary rounds, led by a geriatrician consultant, will be conducted to discuss cases with more complex medical issues and needs, and to monitor the seniors’ progress.

## 5. Conclusions

Singapore faces significant challenges with regard to its ageing population. It is tackling these challenges purposefully through national public policy as well as regional initiatives, backed by state, charity and private funding. Many of these measures are innovative, and represent a deliberate, systematic and proactive approach to the promotion of healthy ageing.
